# The relationship between otitis media and gastroesophageal reflux disease: A 2-sample Mendelian randomization analysis

**DOI:** 10.1097/MD.0000000000044848

**Published:** 2025-10-17

**Authors:** Xinyu Wang, Changhong Yan, Haoyang Yan

**Affiliations:** a Affiliated Baotou Clinical College of Inner Mongolia Medical University, Baotou, China; b Department of Traditional Chinese Medicine, Hunan University of Chinese Medicine, Hunan, China.

**Keywords:** gastroesophageal reflux disease, otitis media, 2-step Mendelian randomization

## Abstract

Existing research suggests a potential link between gastroesophageal reflux disease (GERD) and Otitis media (OM). Nevertheless, the specifics of this correlation are not clearly defined. In order to explore the genetic link between GERD and OM, this study engaged a 2-sample Mendelian randomization methodology. Initial steps comprised careful selection of instrumental variables, namely the single-nucleotide polymorphisms (SNPs), following strict quality control guidelines. The selected SNPs underwent MR analyses involving inverse variance weighting, MR-Egger, weighted median, and simple mode. Horizontal pleiotropy was checked through MR-Egger intercept and the inverse variance weighted tests. To probe the robustness of the observed link and certify result reliability, a leave-one-out analysis was carried out. Using the inverse variance weighted approach, higher genetic liability to GERD was associated with increased risk of acute suppurative OM, chronic suppurative OM, and suppurative and unspecified OM. Concordant evidence was obtained with the weighted median and MR-Egger estimators. Across data sources, heterogeneity tests were not significant (*P* > .05), and the MR-Egger intercept did not indicate directional pleiotropy (*P* > .05), suggesting that horizontal pleiotropy is unlikely to bias the causal estimates. Leave-one-out analyses showed negligible changes when any single SNP was removed, supporting robustness.

## 1. Introduction

Otitis media (OM) is a common and recurring disease primarily among children, often requiring hospitalization and antibiotics.^[1,2]^ Predominantly diagnosed early in life, studies show roughly 90% of children suffer from an exudative form of OM before hitting 4 years, with around 40% facing 6 or more middle ear infections by the age of 7.^[3,4]^ OM is systematically divided into 3 types: acute OM, OM with effusion (OME) developed by ~26% of acute OM patients who encounter constant middle ear effusion lasting for over 3 months, and chronic suppurative OM (CSOM) recognized by a purulent discharge lasting for more than 6 weeks.^[5]^ Notably, about 60% of chronic OM patients are prone to hearing loss, and severe complications such as facial paralysis, cholesteatoma, and brain abscess may occur.^[6]^ This imposes substantial health and financial encumbrances on patients and their families.^[7,8]^

Gastroesophageal reflux disease (GERD) is characterized by the backflow of stomach contents into the esophagus or mouth leading to discomfort, primarily manifesting as acid reflux and heartburn.^[9,10]^ Some research posits GERD as a possible risk factor for OM.^[11,12]^ This association might be due to anatomical factors in children, where a shorter Eustachian tube (ET) may mediate GERD content reflux into the middle ear, prompting disease onset.^[13]^ Concurrently, a segment of observational studies has identified overlaps in patients diagnosed with pediatric GERD and OME.^[14]^ Nonetheless, intricate pathophysiological processes have yielded inconsistent results, and some studies found no connection between these conditions.^[15]^ Consequently, the correlation between OME and GERD necessitates further extensive research. Prior studies have encountered various constraints, including potential confounding factors, reverse causality, and extensive consumption of human and financial resources, among other issues. On the other hand, MR studies use genome-wide association studies (GWAS) and other databases to explore the genetically driven causal relationship between outcome and exposure, thus evading such limitations.

## 2. Materials and methods

### 2.1. Study design description

**Figure 1.**
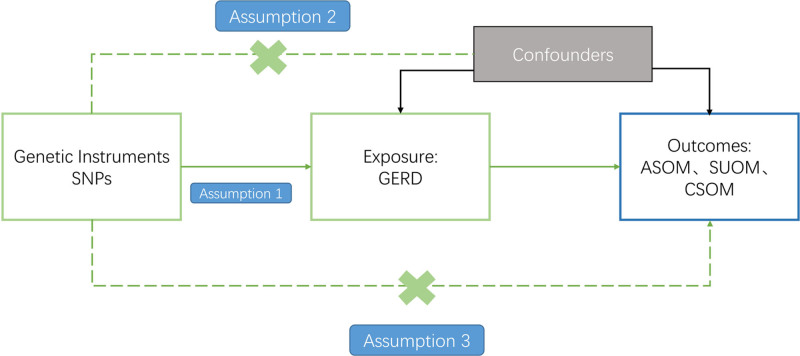
Overall design of the study.

Three critical assumptions are essential for Mendelian randomization (MR) analyses to be satisfactory.^[16,17]^ The first assumption necessitates that there is a significant association between the genetic variables and exposure. Second, the genetic variation used as an instrumental variable for exposure should not be associated with any other confounding variables. Finally, the influence of the genetic variation on the outcome can be affected only through exposure, not by any independent factors.^[18,19]^ The comprehensive layout of this study is demonstrated in Figure 1. The research utilizes a 2-sample MR design to investigate the underlying cause-and-effect relation between suppurative OM and gastro-esophageal reflux disease. GERD was identified as the exposure factor in this study. The outcome measure in this study is suppurative OM, a broad category encompassing acute suppurative OM (ASOM), CSOM, and other types of unclassified OM.

### 2.2. Data sources

Public data from the European Bioinformatics Institute database provided the GWAS summary data for GERD.^[20]^ With informed consent, the study incorporated 129,080 European cases and 473,524 European controls. The outcome data for the research were sourced from the Finnish Biobank (https://r7.risteys.finngen.fi/).^[21]^ The respective GWAS summary data for suppurative and unspecified OM (SUOM), ASOM, and CSOM were also obtained. The data included in this study are derived from 3 contemporary OM studies that included 16,008, 10,987, and 2262 cases, respectively, along with 384,654 controls. The results are shown in Table 1. The 2 populations are largely divergent, which significantly minimizes potential bias.

**Table 1:** Information of different GWAS data.

Exposure/Outcome(s)	Study	Source type	Sample size		Ancestry
			ncase	ncontrol	
Exposure					
GERD	Ong JS	Independent research	129,080	473,524	European
Outcome					
Suppurative and unspecified otitis media	FinnGen	Biobanking data	16,008	384,654	European
Acute suppurative otitis media	FinnGen	Biobanking data	10,987	384,654	European
Chronic suppurative otitis media	FinnGen	Biobanking data	2262	384,654	European

All datasets are predominantly European; exposure and outcome cohorts are nonoverlapping.

GWAS = genome-wide association studies.

### 2.3. Selection of genetic instrumental variables

To guarantee independence and high correlation between exposure factors and outcome variables, we chose instrument variables marked by single-nucleotide polymorphisms (SNPs) with genome-wide importance. Setting our standards according to the comprehensive data from the 1000 Genomes Project, we assigned a genome-wide significance level to gastroesophageal reflux disease at *P* < 5 × 10^−8^, and the correlation discrepancy parameter was fixed at 0.001. Genetic distance was limited to 10,000 kb, excluding any SNPs that had symmetrical structures. In the end, we filtered out SNPs without connection impacts from the GERD dataset. To ensure accurate alignment between exposure and outcome datasets, SNP harmonization was performed using the “TwoSampleMR” package in R. This process involved aligning effect alleles across datasets, removing palindromic SNPs with ambiguous strand orientation, and checking for allele mismatches. Ambiguous SNPs that could not be reliably harmonized were excluded from the analysis. This harmonization procedure ensured consistency in allele orientation and minimized potential strand misalignment bias across the MR analyses.

### 2.4. Statistical analyses

The present research implemented an MR evaluation, employing methodologies such as inverse variance weighting, MR-Egger, and weighted median.^[22,23]^ The threshold was set at an F-value > 10,^[24]^ the statistical strength being computed based on the equation (Beta/se)².^[25]^

### 2.5. Sensitivity analysis

We assessed heterogeneity using 2 methods: the inverse variance weighted (IVW) method and the weighted linear regression (MR-Egger) method.^[22,23]^ A *P*-value below .05 suggests the presence of heterogeneity among SNPs, whereas a *P*-value above .05 indicates homogeneity. To evaluate the influence of individual SNPs on causal associations, we performed sensitivity analyses using a case-by-case exclusion approach. To assess multiplicity, we employed the MR pleiotropy test function, whereby a *P*-value below .05 indicated the presence of multiplicity, while a *P*-value above .05 indicated its absence. Finally, we performed the MR Steiger directionality test to assess whether genetic instruments supported a causal flow from GERD to each outcome phenotype (SUOM, ASOM, CSOM), and we repeated the test in the reverse direction to probe potential reverse causality.

## 3. Results

### 3.1. Genetic instrumental variables for exposures

Table S1, Supplemental Digital Content, https://links.lww.com/MD/Q434 lists 71 SNPs that were significantly associated with GERD based on GWAS. These SNPs were initially considered as potential instrumental variables for the MR analysis. After harmonization, a slightly reduced number of SNPs were retained for the final analysis due to the exclusion of ambiguous variants, such as those with allele mismatches or palindromic structures with non-inferable strands (Table S1, Supplemental Digital Content, https://links.lww.com/MD/Q434). To assess the strength of each instrument, F-statistics were calculated for the SNP–exposure associations. All retained SNPs had F-statistics >10, indicating that they were strong instruments and unlikely to introduce weak instrument bias. The F-statistics for all included SNPs are provided in Table S2, Supplemental Digital Content, https://links.lww.com/MD/Q434.

### 3.2. Causal relationship between GERD and OM

**Figure 2.**
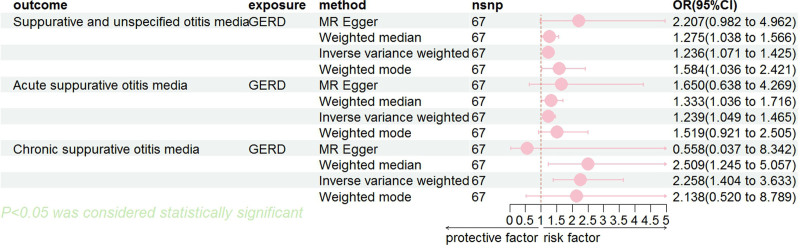
Mendelian randomization primary analysis results.

For MR analysis, we utilized the methods of the IVW model, MR-Egger model, Weighted Median model, and Weighted mode. The study results suggested a potential causal association between genetically predicted GERD and increased risk of ASOM. The IVW method yielded an odds ratio (OR) of 1.24 (95% CI: 1.05–1.46, *P* = .012), while the MR-Egger method produced an OR of 1.65 (95% CI: 0.64–4.27, *P* = .305). Similarly, for CSOM, GERD appeared to be a possible risk factor with an IVW-estimated OR of 2.26 (95% CI: 1.40–3.63, *P* = .001), although the MR-Egger result was nonsignificant (OR = 0.56, 95% CI: 0.04–8.34, *P* = .674). For SUOM, the IVW method supported a causal association (OR = 1.24, 95% CI: 1.07–1.42, *P* = .004), whereas the MR-Egger estimate was borderline significant (OR = 2.21, 95% CI: 0.98–4.96, *P *= .060). However, the MR-Egger intercept tests in all models were nonsignificant (*P* > .05), indicating no strong evidence of directional pleiotropy. These findings strengthen the reliability of the IVW estimates while also underscoring the need for cautious interpretation of MR-Egger results, particularly when estimates are imprecise or inconsistent (Table S3, Supplemental Digital Content, https://links.lww.com/MD/Q434). Table 2 and Figure 2 showcase the outcomes of the primary causal analyses detailed above.

**Table 2:** Main results of MR analysis.

Exposure	Outcome	Method	nsnp	*B*	Se	*P*-value
GERD	Acute suppurative otitis media	MR-Egger	67	0.501	0.485	.305
GERD	Acute suppurative otitis media	Weighted median	67	0.288	0.129	.025
GERD	Acute suppurative otitis media	Inverse variance weighted	67	0.215	0.085	.012
GERD	Acute suppurative otitis media	Weighted mode	67	0.418	0.255	.106
GERD	Chronic suppurative otitis media	MR-Egger	67	-0.584	1.380	.674
GERD	Chronic suppurative otitis media	Weighted median	67	0.920	0.358	.010
GERD	Chronic suppurative otitis media	Inverse variance weighted	67	0.815	0.243	.001
GERD	Chronic suppurative otitis media	Weighted mode	67	0.760	0.721	.296
GERD	Suppurative and unspecified otitis media	MR-Egger	67	0.792	0.413	.060
GERD	Suppurative and unspecified otitis media	Weighted median	67	0.243	0.105	.021
GERD	Suppurative and unspecified otitis media	Inverse variance weighted	67	0.212	0.073	.004
GERD	Suppurative and unspecified otitis media	Weighted mode	67	0.460	0.217	.038

*B* denotes the log odds ratio per 1 standard deviation increase in genetic liability to GERD; Se is the standard error of *B*. Odds ratios and 95% confidence intervals can be obtained as OR = exp(*B*) and 95% CI = exp(*B* ± 1.96 × Se). *P*-values are 2-sided. nsnp is the number of independent instrumental variants retained after clumping and harmonization.

GERD = gastroesophageal reflux disease, MR = Mendelian randomization.

### 3.3. Results of heterogeneity and sensitivity analyses

**Figure 3.**
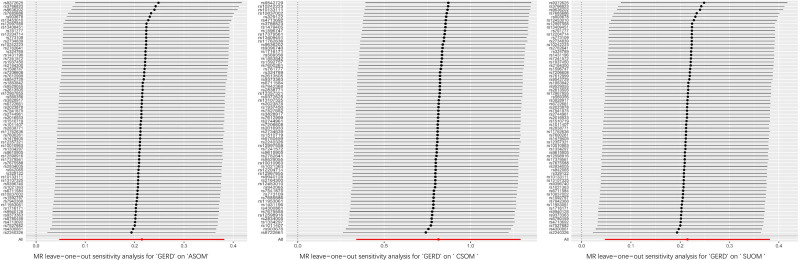
Leave-one-out of the Mendelian randomization (MR) analysis. Each point represents the IVW estimate recalculated after removing 1 instrument. Stability across points indicates that no single SNP drives the result. IVW = inverse variance weighted, SNP = single-nucleotide polymorphism.

**Figure 4.**
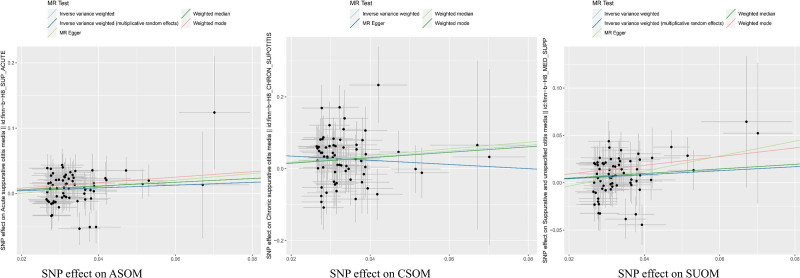
Scatter plots of the Mendelian randomization (MR) analysis. Each point is a single-variant effect; lines show IVW, weighted median, and MR-Egger fits, whose slopes equal the causal estimates. Line thickness reflects estimator weighting. IVW = inverse variance weighted.

Sensitivity analyses did not reveal a significant heterogeneity or evidence of horizontal pleiotropy in the associations between GERD and SUOM, CSOM, or ASOM, as indicated by nonsignificant Cochran Q tests and MR-Egger intercepts (*P *> .05 for all; see Table 3). Similar results were observed across the majority of diagnostic tests. Furthermore, leave-one-out analyses demonstrated that no single SNP exerted a disproportionate influence on the overall causal estimates (Fig. 3), supporting the robustness of the findings. Collectively, these results indicate that the observed associations between GERD and various subtypes of OM are consistent and unlikely to be driven by pleiotropy or heterogeneity (Fig. 4). To address inflated type I error from evaluating 3 related OM phenotypes, we applied a Benjamini–Hochberg false discovery rate (FDR) procedure to the prespecified primary IVW tests across SUOM, ASOM, and CSOM. After FDR control at 5%, all associations remained significant with FDR-adjusted *P*-values of .006 for SUOM, .012 for ASOM, and .003 for CSOM (Table S4, Supplemental Digital Content, https://links.lww.com/MD/Q434). The point estimates and directions were unchanged, indicating that the observed signals are unlikely to reflect multiplicity alone. Because IVW was designated a priori as the primary causal estimator, secondary models (MR-Egger, weighted median, weighted mode) were not further adjusted for multiplicity and were used as robustness checks. Given the shared pathophysiology and correlation among OM subtypes, FDR offers an appropriate balance between error control and power. Finally, applying the MR Steiger test, we oriented the effect from GERD to SUOM, ASOM, and CSOM and found no support for reverse causation; the direction was consistently upheld (Table 4).

**Table 3:** Sensitivity analysis of GERD.

Outcome	Heterogeneity test		Horizontal pleiotropy
	*Q P*val (IVW)	*Q P*val (MR-Egger)	
SUOM	.611	.648	0.159
ASOM	.595	.573	0.551
CSOM	.672	.675	0.307

Heterogeneity was assessed using Cochran *Q* under the IVW and MR-Egger models. Horizontal pleiotropy was evaluated using the MR-Egger intercept. *P*-values are 2-sided.

ASOM = acute suppurative otitis media, CSOM = chronic suppurative otitis media, GERD = gastroesophageal reflux disease, IVW = inverse variance weighted, MR-Egger = Mendelian randomization Egger regression, SUOM = suppurative and unspecified otitis media.

**Table 4:** The results of the MR Steiger test.

Exposure	Outcome	*R*^2^ for exposure	*R*^2^ for outcome	Correct causal direction	*P*_steige_
GERD	ASOM	4.30 × 10^−3^	1.74 × 10^−4^	TRUE	6.08 × 10^−145^
GERD	CSOM	4.30 × 10^−3^	1.85 × 10^−4^	TRUE	7.71 × 10^−141^
GERD	SUOM	4.30 × 10^−3^	1.76 × 10^−4^	TRUE	1.21 × 10^−145^

Steiger direction indicates the orientation favored by the MR Steiger test.

*R*^2^, the variance of phenotype explained by genetic instruments.

ASOM = acute suppurative otitis media, CSOM = chronic suppurative otitis media, GERD = gastroesophageal reflux disease, MR = Mendelian randomization, SUOM = suppurative and unspecified otitis media.

## 4. Discussion

Current assessments of observational studies suggest that the incidence of GERD in children suffering from OM could exceed the general prevalence in children. Multiple studies have potentially linked GERD to OM.^[26]^ However, OM entails diverse disease categorizations, such as ASOM, CSOM, and OME, individualized by onset time and individual conditions.^[27]^ Given the limited data, we treated ASOM, CSOM, and SUOM as separate outcomes because they reflect clinically and biologically meaningful differences. ASOM denotes an acute suppurative process typically precipitated by nasopharyngeal inflammation and dysfunction of the ET. CSOM represents chronic suppuration characterized by tympanic membrane perforation, granulation tissue, and biofilm-mediated persistence with distinct microbial profiles. SUOM is marked by middle ear effusion without signs of acute infection and most often presents with hearing impairment. It is primarily an inflammatory and mechanical disorder in which ET dysfunction and cytokine-driven exudation are central, while bacterial infection is frequently secondary or absent. Although SUOM overlaps conceptually with other entities, including it broadens outcome coverage and enables sensitivity analyses. Because these subtypes differ in temporal course, tissue remodeling, and host and pathogen interactions, GERD-related pathways may influence disease initiation (ASOM and SUOM) differently from disease persistence (CSOM). Analyzing them separately avoids diluting heterogeneous effects, improves clinical interpretability, and allows a more precise appraisal of the potential role of GERD in each OM phenotype while meeting the MR requirement for clearly defined outcomes. Against this backdrop, we focused on the most clinically prevalent suppurative phenotypes and evaluated SUOM, ASOM, and CSOM as outcomes to test whether genetic liability to GERD causally influences OM risk. This design leverages MR to mitigate confounding and reverse causation that often challenge conventional epidemiology.

Our MR results extend prior observational reports linking gastroesophageal reflux and OM by leveraging genetic instruments to reduce confounding and reverse causation. Whereas earlier cohort and case-control studies were limited by exposure misclassification and shared environmental risks, the present estimates, consistent across multiple MR estimators and retained after FDR control, support a causal contribution of GERD liability to susceptibility for SUOM, ASOM, and CSOM. These findings align with, and add causal inference to, the existing clinical literature.

Several complementary pathways may explain a causal contribution of GERD to OM. Reflux-related inflammation in the nasopharynx can impair ET function, predisposing the middle ear to negative pressure and secondary infection; this vulnerability is accentuated in children because the ET is shorter, more compliant, and more horizontally oriented.^[28]^ Clinically, higher rates of bilateral OM with effusion have been reported among individuals with reflux, consistent with a reflux–ET dysfunction axis that facilitates middle ear disease.^[29]^ Micro-aspiration of gastric contents can further amplify risk through direct tissue effects and altered airway defence. Acidic refluxate can damage the upper-airway and middle ear mucosa, promote edema, and disrupt epithelial barrier integrity, thereby lowering the threshold for secondary infection.^[30]^ In addition, pepsin and related proteolytic enzymes present in refluxate may impair mucociliary clearance and ventilation of the middle ear, disturb local microbial ecology, and sustain mucosal immune activation, collectively creating conditions favorable to suppurative OM.^[31]^ Although MR cannot adjudicate among these mechanisms, the convergence of our genetic findings with these biologically plausible pathways supports the credibility of a directional effect from GERD to OM.^[13]^

This research holds several advantages. Primarily, the application of MR to uncover the cause–effect relationship between GERD and ASOM/CSOM for the first time. Our interpretation rests on the core MR assumptions: relevance (instruments associate with GERD), independence (instruments are not related to confounders), and exclusion restriction (instruments affect outcomes only through GERD). To support relevance and validity, we selected genome-wide significant variants, applied LD clumping, removed palindromic and putative confounder-linked SNPs, and harmonized alleles. We report instrument strength metrics (F-statistics and variance explained) in Table S1, Supplemental Digital Content, https://links.lww.com/MD/Q434. Moreover, the pooled GERD GWAS data derived from 2 independent European population further reduced possible bias from overlapping exposure. Ultimately, the primary signals were directionally consistent across complementary estimators, MR Steiger testing oriented the effect from GERD to each outcome rather than the reverse, and diagnostics did not indicate material violations of MR assumptions. Cochran *Q* indicated no material heterogeneity, and the MR-Egger intercept showed no evidence of directional pleiotropy. After applying Benjamini–Hochberg FDR control across the 3 prespecified IVW tests, all associations remained statistically significant with unchanged magnitude and direction. Leave-one-out analyses further showed that no single SNP unduly influenced the results.

Nevertheless, acknowledging this study’s limitations is essential. The statistical outcomes of the MR study showed that the *P*-values from the IVW method were all lower than .05. Yet, all OR values were little above 1, and the variance was insignificant due to potential yet unknown confounding influences. Despite this, the leave-one-out method showed steady results, indicating that even if such confounding variables existed, their influence on the experiment’s primary findings was relatively limited. Concerning the selection of the study population, an absence of available GWAS data from Asian populations prevented the study from being conducted in that demographic. However, the results may not completely apply to subjects from other populations. If relevant GWAS data from Asian demographics become available in the future, we will carry out correlation analyses with that data. In the meantime, since the pooled GWAS data study population comprised significantly of children, these outcomes might not be applicable to the adult population.

In summary, by indicating a causal contribution of GERD liability to OM susceptibility, our findings highlight reflux control as a potentially modifiable upstream target in individuals at risk of recurrent or chronic middle ear disease. Prospective studies that integrate reflux phenotyping with otologic outcomes, together with mechanistic work on upper-airway mucosal immunity, are warranted to refine risk stratification and test intervention effects.

## 5. Conclusions

In conclusion, utilizing genetic data, our study applied a 2-sample bidirectional MR analysis to illuminate the causal ties between GERD and ACOM/CSOM. A deeper comprehension of the cause–effect relationship between the two could foster advancement in health education and routine colorectal cancer screenings for ACOM/CSOM patients. In addition, it could enable early interventions, potentially impacting ACOM/CSOM prevalence control positively. To further verify and elucidate the biological mechanics behind GERD and ACOM/CSOM association, future research should consider utilizing additional experimental designs.

## Acknowledgments

We are grateful to all the students and teachers who took part in the work of writing the article.

## Author contributions

**Conceptualization:** Xinyu Wang, Changhong Yan.

**Data curation:** Changhong Yan.

**Software:** Haoyang Yan.

**Writing – original draft:** Xinyu Wang.

**Writing – review & editing:** Xinyu Wang.

## Supplementary Material


